# Identification of a novel gene signature for the prediction of recurrence in HCC patients by machine learning of genome-wide databases

**DOI:** 10.1038/s41598-020-61298-3

**Published:** 2020-03-10

**Authors:** Jie Shen, Liang Qi, Zhengyun Zou, Juan Du, Weiwei Kong, Lianjun Zhao, Jia Wei, Ling Lin, Min Ren, Baorui Liu

**Affiliations:** 1 Comprehensive Cancer Centre of Drum Tower Hospital, Medical School of Nanjing University, Clinical Cancer Institute of Nanjing University, Nanjing, 210008 Jiangsu Province China; 2 Shanghai Biotecan Pharmaceuticals Co., Ltd., Pudong New District, Shanghai, China

**Keywords:** Tumour biomarkers, Cancer genomics, Hepatocellular carcinoma

## Abstract

Hepatocellular carcinoma (HCC) is a common malignant tumor in China. In the present study, we aimed to construct and verify a prediction model of recurrence in HCC patients using databases (TCGA, AMC and Inserm) and machine learning methods and obtain the gene signature that could predict early relapse of HCC. Statistical methods, such as feature selection, survival analysis and Chi-Square test in R software, were used to analyze and select mutant genes related to disease free survival (DFS), race and vascular invasion. In addition, whole-exome sequencing was performed on 10 HCC patients recruited from our center, and the sequencing results were compared with the databases. Using the databases and machine learning methods, the prediction model of recurrence was constructed and optimized, and the selected mutant genes were verified in the test group. The accuracy of prediction was 74.19%. Moreover, these 10 patients from our center were used to verify these mutant genes and the prediction model, and a success rate of 80% was achieved. Collectively, we discovered recurrence-related genes and established recurrence prediction model of recurrence for HCC patients, which could provide significant guidance for clinical prediction of recurrence.

## Introduction

Hepatocellular carcinoma (HCC) is a common malignant tumor in China, which ranks the third in morbidity and the second in mortality. Its morbidity is usually associated with specific risk factors, including infections with HBV and HCV, high alcohol intake, obesity and consumption of aflatoxin-containing food^1^. With the development of the second-generation sequencing techniques increasing research on HCC has been conducted on the molecular level. In 2014, Totoki *et al*.^2^ have reported the whole-genome sequencing of 608 HCC patients from Asia and Europe. In 2015, Schulze *et al*.^3^ have reported the whole-genome sequencing of 243 HCC patients from Europe and America. In 2016, Fujimoto *et al*.^4^ have reported the whole-genome sequencing of 300 HCC patients from Japan. The molecular blueprint of HCC including somatic mutation, mRNA expression, methylation and miRNA regulation has been gradually outlined, which could be used for the diagnosis, treatment, and prediction of recurrence and survival of liver cancer patients. In 2017, TCGA working group^5^ has systematically analyzed the sequencing results of the whole exome of more than 360 HCC patients in TCGA database and compared these data with other published HCC sequencing samples. Various statistical methods, related classification and clustering algorithms of machine learning have been used. TERT, TP53, CTNNB1, AXIN1, ARID1A, ARID2, RB1, ALB, APOB, PTEN, CDKN2A, DOCK2^6–15^ and other somatic cells with significantly mutant genes (SMGs) and driver mutation have been identified. These findings have been rapidly applied as potential therapeutic targets and prognostic indicators in clinical practice.

However, the high cost of whole-exome sequencing and whole-genome sequencing limits its use in clinical practice. Actually, patients often can afford the commercial panels launched by gene sequencing companies. Those panels are much cheaper than whole-genome or whole-exome sequencing. However, many of these commercial panels contain a combination of genes in various cancers. Therefore, a more accurate and economical panel of genes is necessary to guide treatment and recurrence prediction for HCC patients.

In the present study, we used a variety of machine learning algorithms to mine the TCGA, AMC and Inserm databases to screen mutant genes related to disease free survival (DFS), race and vascular invasion and so on. The whole-exome sequencing was performed in 10 patients from our hospital to evaluate the clinical operability of the candidate genes.

## Results

### Analysis of DFS-related genes

**Figure 1:**
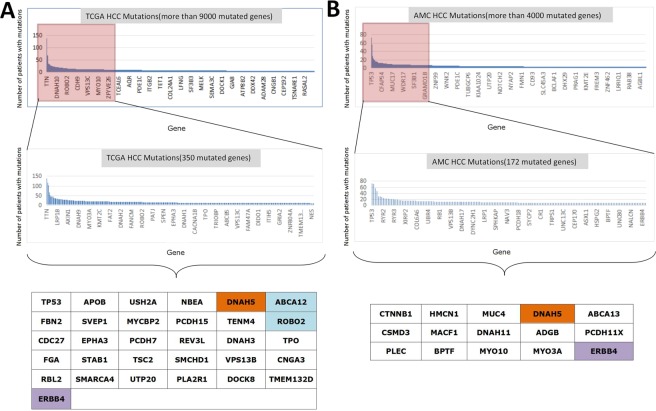
(**A**) A total of 31 genes with significant differences in DFS were selected from the TCGA database. Brown color indicates that the gene is also statistically different in AMC database. Blue color indicates that the gene is also statistically different in AMC database, while it is not a highly frequent mutation. Purple color shows that the gene is also statistically different in AMC database, while such difference is opposite. (**B**) A total of 15 genes with significant differences in DFS were selected from AMC database. Brown color indicates that the gene is also statistically different in TCGA database. Purple color shows that the gene is also statistically different in AMC database, while such difference is opposite.

From the data analysis of public databases, we screened some potential DFS-related mutant genes. A total of 31 genes with significant differences in DFS were selected from the TCGA database (Fig. 1A). Moreover, 15 genes with significant differences in DFS were selected from the AMC database (Fig. 1B). However, the repeatability of these mutant genes was poor between different databases, while only DNAH5, ABCA12, ROBO2 and ERBB4 remained significant. By analyzing these four genes, we found that DNAH5 was mutated to cause a poorer DFS in both TCGA and AMC databases, while an opposite conclusion was drawn from ERBB4. For ABCA12 and ROBO2, although both mutations in TCGA and AMC databases led to a poorer DFS, the total number of mutation cases of ABCA12 and ROBO2 was too small. This finding suggested that the mutation frequency was low, and these genes needed to be verified in a larger number of samples. Fig. S1 illustrates the KM survival curve.

### Analysis of race-related genes

The 356 HCC cases in the TCGA database contained complete race information. Asian people and non-Asian people have different causes of disease and different genetic backgrounds. Therefore, the database and the screened risk-related genes might be different^5,16^. To study whether mutant genes were different among different races, we specifically divided the race into two categories, namely Asian (158 cases) and non-Asian (198 cases). The Boruta algorithm in R software was used to preliminarily screen the genes with mutation differences between Asian and non-Asian HCC patients from the top 350 mutant genes of TCGA database. Our results showed that 12 mutant genes might have race difference among the 350 mutant genes. Next, by Fisher’s test or Pearson’s test, P < 0.05 was used to select the genes with different mutations between Asian and non-Asian patients. Several mutant genes, such as DNAH5, MKI67, KRT10, COL6A3 and FLG, were found (Table 1). AMC and Inserm databases did not list the race information of patients, so relevant analysis was not conducted.

**Table 1:** Race-related gene analysis.

Gene	Mutation Type	Asian	Non-Asian	P
DNAH5	Mutation	9	1	0.006153
	Wild Type	149	197	
MKI67	Mutation	9	1	0.006153
	Wild Type	149	197	
KRT10	Mutation	7	1	0.02423
	Wild Type	151	197	
COL6A3	Mutation	1	13	0.009685
	Wild Type	157	185	
DNAH3	Mutation	8	3	0.06748
	Wild Type	150	195	
CACNA2D1	Mutation	7	2	0.08371
	Wild Type	151	196	
PIK3CA	Mutation	8	3	0.06748
	Wild Type	150	195	
PCDHB16	Mutation	9	3	0.06063
	Wild Type	149	195	
DMD	Mutation	12	6	0.08735
	Wild Type	146	192	
EPB41L3	Mutation	8	3	0.06748
	Wild Type	150	195	
AHNAK	Mutation	10	8	0.4619
	Wild Type	148	190	
FLG	Mutation	16	8	0.03914
	Wild Type	142	190	

### Analysis of vascular invasion-related genes

Several authoritative literatures have reported that among the factors related to the prognosis of HCC patients, tumor size and vascular invasion are the main factors, so we separately listed vascular invasion^17,18^. The 306 HCC patients in the TCGA database had detailed vascular invasion information, including major vascular invasion, microvascular invasion and non-vascular invasion. AMC database contained vascular invasion information of 231 HCC patients. Inserm database contained vascular invasion information of 236 HCC patients. For the convenience of the study, we divided vascular invasion into two categories, including vascular invasion and non-vascular invasion.

Similarly, Boruta algorithm, a feature selection algorithm in R software, was used to preliminarily screen the potential mutant genes that might have differences in vascular invasion from the 350 mutant genes of TCGA database, 172 mutant genes of AMC database and 211 mutant genes of Inserm database (Table 2). Next, by Fisher’s test or Pearson’s test, P < 0.05 was used to select the genes with different mutations between vascular invasion and non-vascular invasion. OBSCN in TCGA database, PLXNA1, MUC12 and BSN in AMC database, and BIRC6, DNAH5, PKHD1, TSC2, KIAA1109 and DYNC1H1 in Inserm database were detected.

**Table 2:** Vascular invasion-related genes.

Gene	Boruta algrithm* (P values)			Fisher’s test and Pearson’s test (P values)		
	TCGA	Inserm	AMC	TCGA	Inserm	AMC
AKAP6	P < 0.05			0.1862	1.0000	0.3251
OBSCN	P < 0.05			0.0210	0.5661	0.5330
TSC2	P < 0.05	P < 0.05		0.1285	0.0317	0.6768
LAMA1	P < 0.05			0.2509	0.7299	0.2885
BIRC6		P < 0.05		0.7260	0.0415	0.3633
DNAH5		P < 0.05		0.8609	0.0171	0.5176
PKHD1		P < 0.05		0.1894	0.0415	0.6734
KIAA1109		P < 0.05		1.0000	0.0415	0.5599
DYNC1H1		P < 0.05		0.2714	0.0232	0.5149
FCGBP		P <0.05		0.5030	0.0735	0.6734
FREM2		P < 0.05		0.1894	0.1289	0.4228
PLXNA1			P < 0.05	0.3550	0.1817	**0.0246**
MUC12			P < 0.05	1.0000	1.0000	**0.0204**
BSN			P < 0.05	0.2371	1.0000	**0.0208**
PLA2G4A			P < 0.05	0.2359	1.0000	0.0506
LAMA2			P < 0.05	0.7700	0.6626	0.1640
PTPRZ1			P < 0.05	0.7405	0.4576	0.06794
CIT			P < 0.05	1.0000	1.0000	0.0866

*Boruta algorithm is a preliminary screening algorithm. P < 0.05 is the preset condition for preliminary screening. The relevant genes screened out do not give specific P values. After preliminary screening, Fisher’s test and Pearson’s test are used for accurate calculation.

Therefore, by analyzing the mutant genes and clinical information of TCGA, AMC and SC databases, we preliminarily screened the DFS-related mutant genes (DNAH5, ABCA12, ROBO2 and ERBB4), race-related mutant genes (DNAH5, MKI67, KRT10, COL6A3 and FLG) and vascular invasion-related genes (OBSCN, TSC2, BIRC6, DNAH5, PKHD1, KIAA1109, DYNC1H1, PLXNA1, MUC12 and BSN). These mutant genes could be used for clinical prediction or verified with sequencing information from Chinese population.

### Whole-exome sequencing of 10 patients in our center and comparison with TCGA, AMC and Inserm databases

**Figure 2:**
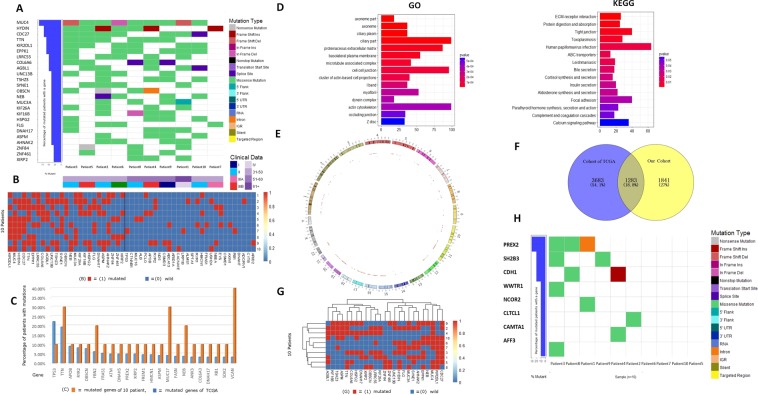
(**A**) Heat maps of somatic cell mutation, stage and age information in 10 patients with HCC; (**B**) left: Highly frequent mutant genes in 10 patients (25 in total). Right: Highly frequent mutant genes in TCGA database (28 in total). Heat maps were generated for the 53 gene mutations in 10 patients. The frequency of TCGA mutations was not high in our 10 patients. (**C**) Comparison of high frequency gene mutations between 10 HCC patients in our center and TCGA database. (**D**) GO and KEGG pathways involved in 10 HCC patients in our center. (**E**) Circos of mutation information in 10 HCC patients. (**F**) Venn diagram for comparison of mutant genes and TCGA mutant genes in 10 HCC patients. (**G**) Clustering heat map of high frequency mutant genes in 10 HCC patients. (**H**) Heat map of driver gene mutation in 10 HCC patients.

The top 20 mutant genes with high mutation frequency found in 360 HCC patients from TCGA database included TP53, TTN, CTNNB1, MUC16, propagated, PCLO, APOB, RYR2, ND5, CSMD3, OBSCN, ABCA13, ARID1A, CACNA1E, LRP1B, XIRP2, ALMS1, SPTA1, RYR1 and HMCN1. Whole-exome sequencing was performed on the tumor tissues collected from the 10 HCC patients (Fig. 2A), with an average sequencing depth of 74. The 25 mutant genes with the highest mutation frequency were mapped into heat map (MUC4, HYDIN, CDC27, TTN, KIR2DL1, EPPK1, LRRC55, COL6A6, AGBL1, UNC13B, TSH23, SYNE1, OBSCN, NEB, MUC3A, KIF26A, KIF16B, HSPG2, FLG, DNAH17, ASPM, AHNAK2, ZNF84, ZNF461 and XIRP2).

After comparison with TCGA and AMC databases (Fig. 2B,C), we found that KIR2DL1 EPPKI, LRRC55, MUC3A and ZNF84 were not apparent in TCGA database. EPPKI, LRRC55, MUC3A, ZNF84 and ZNF461 were not apparent in AMC database. GO enrichment and KEGG analysis were shown in Fig. 2D,E. TP53, CTNNB1, AXIN1, ARID1A, ARID2, RB1, ALB, APOB, PTEN, CDKN2A and DOCK2 were mutant genes with high frequency in most databases, while the corresponding number of mutation in the 10 patients collected from our center was 1, 0, 1, 0, 0, 2, 1, 1, 2, 0, 0 and 0, respectively.

Among all detected mutations, DNAH5 and ABCA12 were found in three patients and two patients, respectively, while mutations of EPHA3, ROBO2 and ERBB4 were not found in these 10 patients. The results suggested that the DFS-related mutant genes screened from the large sample database might be undetectable in the small sample population due to its low mutation frequency, thus losing the universal value of predicting recurrence. Figure 2F shows the Venn diagram for comparison of mutant genes in TCGA and mutant genes in 10 HCC patients. Figure 2G shows the Clustering heat map of high-frequency mutant genes in 10 HCC patients.

The accumulation of somatic cell mutations leads to the occurrence and development of tumors. For the above-mentioned somatic cell mutations, we selected the driver genes in 10 samples by comparing them with the driver genes listed by Cancer Gene Census: PREX2, SH2B3, CDH1, WWTR1, NCOR2, CLTCL1, CAMTA1 and AFF3 **(**Fig. 2H).

We compared genome-wide/exome sequencing data from three independent databases. Similarly, the clinical information collected from these databases (including age, gender, race, vascular invasion/cancer thrombus, DFS, OS and so on) should be utilized whenever possible. Boruta algorithm was used for feature gene screening. The selected genes were as follows: OBSCN, TSC2, BIRC6, DNAH5, PKHD1, KIAA1109, DYNC1H1, PLXNA1, MUC12 and BSN. It was found that only OBSCN was overlapped with the 25 highly frequent mutant genes detected in 10 samples collected from our center. At the same time, we observed that the mutation frequency of KIR2DL1 was higher in 10 HCC patients collected from our center and AMC database, while the gene mutation of KIR2DL1 was not detected in TCGA and Inserm databases, considering the geographical and ethnic differences of this gene mutation.

### Construction of a model for predicting recurrence of mutant genes

#### Decision tree model

**Figure 3:**
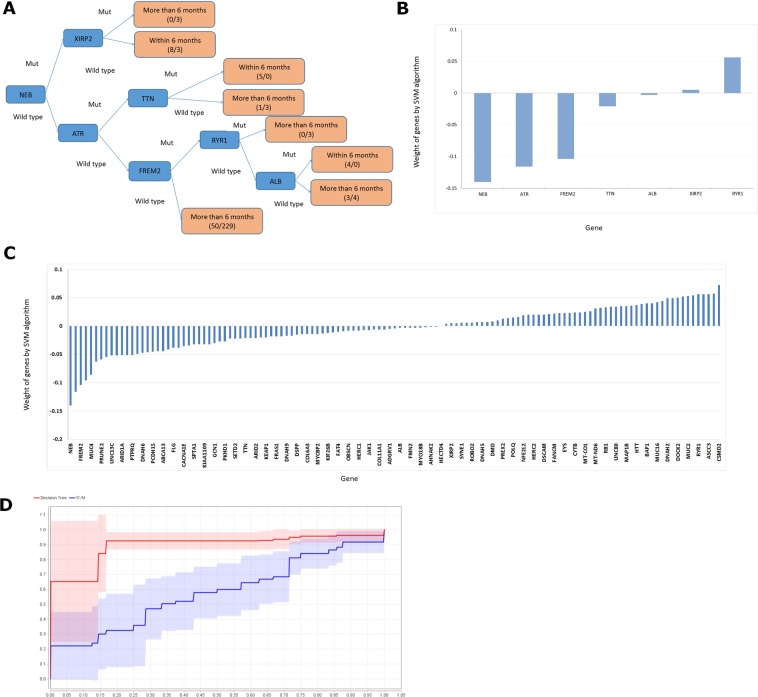
(**A**) The flow of decision tree model; (**B**) The prediction weight of node genes in the decision tree; (**C**) The weight of each gene analyzed by SVM Model; (**D**) the ROC curves of the decision tree model and the SVM model are compared.

We extracted 315 HCC patients with complete DFS data from the TCGA database. According to the ratio of 1:9, these cases were randomly divided into the model group and test group, respectively. The first 12 mutant genes, the first 50 mutant genes, the first 100 mutant genes, the first 200 mutant genes and the first 350 mutant genes were used for decision tree modeling. After numerous tests, the prediction accuracy of the decision tree model for the first 127 mutant genes (Table S1) was the highest (74.19%), and the area under the ROC curve (AUC) was 0.750 (Table S2). The generated decision tree model was illustrated in Fig. 3A,B. Moreover, we validated the results using the AMC database, and the accuracy rate reached 70.41% (Table S2).

#### Support-vector machine (SVM) model

We extracted 315 HCC patients with complete DFS data from the TCGA database. According to the ratio of 1:9, these cases were randomly divided into the model group and test group, respectively. The first 12 mutant genes, the first 50 mutant genes, the first 100 mutant genes, the first 200 mutant genes and the first 350 mutant genes were used for SVM modeling. After numerous tests, the prediction accuracy of the decision tree model for the first 127 mutant genes was the highest (80.65%), while the AUC was only 0.595 (Table S3). Figure 3C shows the weight of each gene. The ROC curves of the decision tree model and SVM model were compared (Fig. 3D). Although the accuracy of the SVM model was higher, the decision tree model was more balanced and more intuitive. Taken together, the SVM model was more abstract.

#### Verification of the test model in 10 HCC patients

We used the decision tree model to test the 10 patients collected from our center. The related genes included NEB, ATR, FREM2, TTN, ALB, XIRP2 and RYR1. Table S4 shows that except for patients 1 and 3, who had the recurrence time of more than 6 months according to the decision tree model, and the rest of the eight patients were all predicted correctly. The agreement rate between the results from the decision tree model and the clinical observation reached 80%.

## Discussion

There are several important databases and studies of whole-genome sequencing and whole-exome sequencing related to somatic cell mutation of liver cancer. Through in-depth studies, many scholars have discussed the detailed roles of TP53^7^, CTNNB1^7^, TERT^8^, ARID1A, RB1, CDKN2A^9,14^, CCND1, AXIN1^9^, ARID2^10^, PTEN^13^ and other common mutant genes^15^ in the occurrence, development, recurrence and prognosis of HCC. Meanwhile, the roles of these genes in WNT, PIK3CA, JAK, mTOR^19^ and other pathways^20^ have been gradually clarified with the accumulation of cases studied by second-generation sequencing. More and more high-quality studies have provided reliable potential targets for the research and development of targeted drugs. However, as a tumor with high heterogeneity, HCC is characterized by rapid progression, poor prognosis, high mortality, and low efficiency of targeted drugs, such as sorafenib and lenvatinib. Although surgery, transcatheter arterial chemoembolization (TACE), radiofrequency ablation, radiotherapy and other therapeutic approaches can control the progress of HCC, generally speaking, HCC is still under exploration in the areas of molecular typing, molecular diagnosis and gene target selection. Although CDH^21^, LDH^22^, NCOR1^23^ and other mutant genes are new biomarkers in HCC subtype classification^24,25^, identification of gene mutations that affect recurrence is complex but will have clinical significance. Meanwhile, various probability theories and linear algebra algorithms can be used for comprehensive analysis.

In the present study, 10 cases of HCC patients in Nanjing Drum Tower Hospital were sequenced by whole-exome sequencing, and highly frequent mutant genes, such as MUC4, HYDIN, CDC27, TTN^26^, COL6A6, SYNE1, NEB, OBSCN, NEB, HSPG2, FLG, DNAH17, ASPM, AHNAK2 and XIRP2, were also detected in the TCGA database. OBSCN was associated with HCC tumor thrombectomy, while CDC27 was associated with recurrence. KIR2DL1, EPPKI, LRRC55, MUC3A and ZNF84 were high-frequency mutations in the samples that we tested in our center but not in TCGA. There might be several reasons for this. Firstly, the current database is mostly based on non-Asian people, while our test was carried out based on Asian people. Asian people and non-Asian people have different causes of disease and different genetic backgrounds. Therefore, the database and the screened risk-related genes are different. On the other hand, we sequenced 10 samples, a small sample size, which might also cause gene deviation. Recently, Fan J’s team has sequenced 159 Chinese patients with HCC and found that the mutation frequency of AXIN, TSC2, SMARCA2, ATRX, KMT2C is higher than that of HBV-related diseases reported by TCGA, while CTNB1, ARID1A and RB1 is lower, suggesting that the mutation spectrum of HBV-related HCC population in China is different from that in Western countries, which is the same in 10 HCC patients in our center^27^. In this study, a large sample base of TCGA was used to construct an HCC recurrence model by machine learning study, which was verified in 10 patients from our center. The agreement rate was 80%, and our data could be used as a reference for clinical prognosis. Meanwhile, due to the randomness of gene mutations, the application of the above-mentioned mutant genes in predicting recurrence, typing and other aspects still needs careful verification.

## Methods

### Data collection

The gene mutation data of HCC in TCGA database collected by cBioportal and the gene mutation data of AMC database were used in the present study. Moreover, the corresponding clinical data were downloaded at the same time, and the HCC cases with insufficient clinical information were removed. Finally, the information of gene mutation and clinical data were integrated. According to the definition of gene mutation in TCGA database, gene mutations were subdivided into several main common variants as follows: missense mutation, nonsense mutation, truncating mutation (including splice, frameshift deletion and frameshift insertion) and inframe (including inframe deletion and inframe insertion). In addition, genomicalterations were also subdivided into copy number alteration (CNA or CNV), SNP, deletion, insertion and so on. In combination with TCGA data analysis, this study did not subdivide specific SNP and CNV, but only studied non-synonymous mutations (Fig. S2).

**Figure 4:**
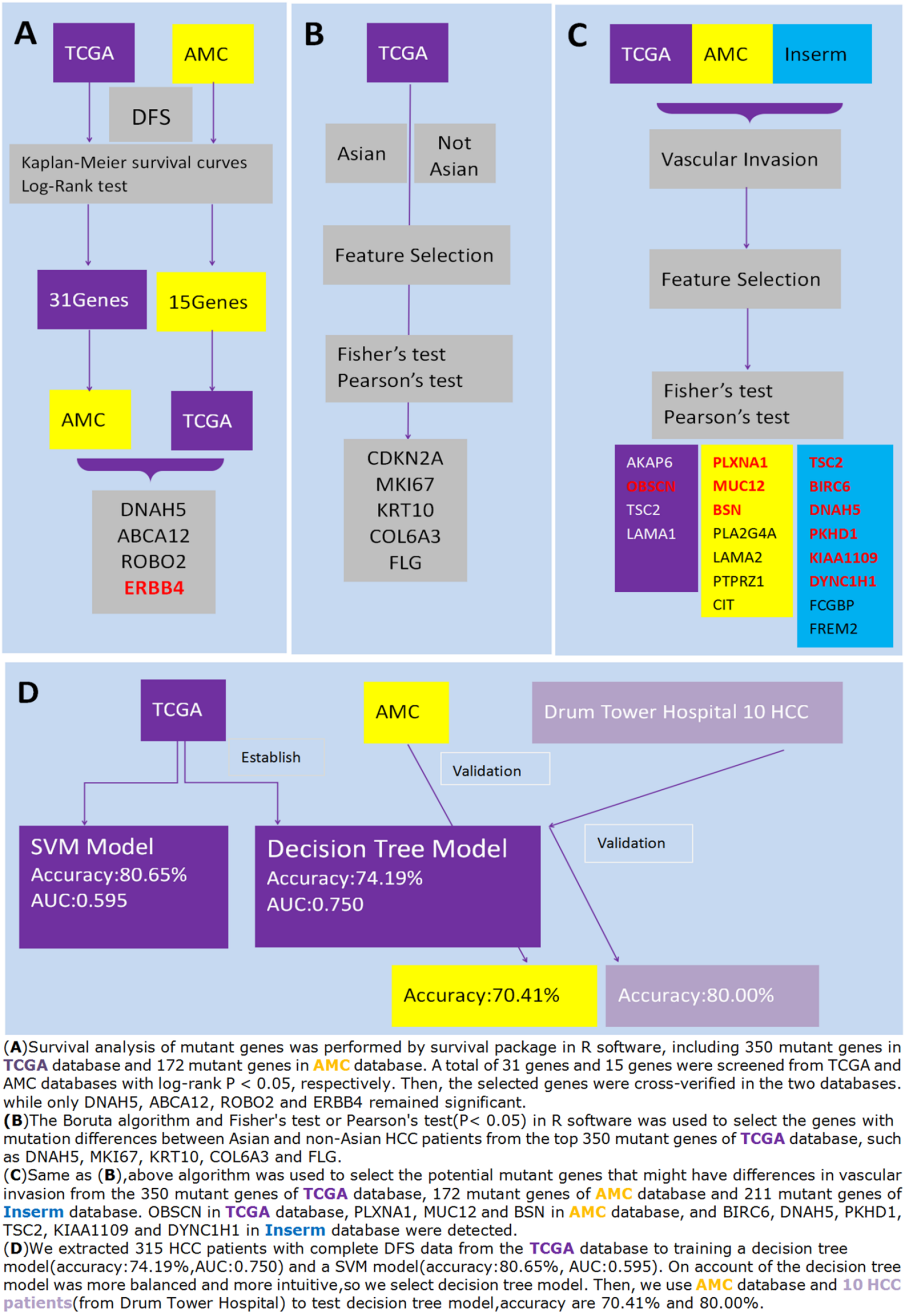
The whole study flow. (**A**) Kaplan-Meier survival analysis and log-rank test were used to screen DFS-related mutant genes from TCGA database and AMC database. Then these genes were cross-verified in TCGA and AMC, and four DFS-related mutant genes were screened out in these two databases; (**B**) Boruta algorithm, Fisher’s test and Pearson’s test were used to screen race (Asian/non-Asian)-associated mutations from TCGA database; (**C**) Boruta algorithm, Fisher’s test and Pearson’s test were used to screen vascular invasion-associated mutations from TCGA, AMC and Inserm database; (**D**) The HCC data in TCGA were used to construct a model for predicting recurrence, and then AMC and 10 HCC patients in our center were used for verification.

Over 9,000 mutant genes were downloaded from TCGA database, and a total of 350 mutant genes apparent in more than eight patients were selected for analysis. Similarly, over 4,000 mutant genes were also downloaded from 231 HCC patients in AMC database, and those genes apparent in more than eight patients were verified. Data from Insrem database were also analyzed. The above-mentioned genes were analyzed using feature selection, survival analysis, Chi-Square test, Fisher exact test and other algorithms. Figure 4 illustrates the whole study flow.

Figures 1A,B, 2C and 3B,C made by Microsoft Office Excel2003/WPS Office2019. Figure 2A,B,D–H made by R3.5.1 and RStudio. Figure 3A,D made by RapidMiner(a software use machine learning to data mining) and Microsoft Office Word2003/WPS Office2019. Figure 4 made by Microsoft Office Word2003/WPS Office2019.

### Analysis of DFS-related genes

First, for TCGA clinical data, cases with missing clinical information were excluded, and the remaining 116 cases had complete information, including age, height, weight, race, family history of tumor classification, operation method, tumor differentiation, AJCC staging, vascular invasion, Child-Pugh score, the degree of liver fibrosis, liver tissue inflammation, and ECOG score. DFS was converted into binary variables with “relapse within 6 months” and “relapse after 6 months”, and the logistic regression in RapidMiner Studio 8.1 was used for data-mining analysis. Similarly, logistic regression was used to analyze the age, gender, BCLC stage, HBV/HCV, cirrhosis grade, vascular invasion, tumor size, Edmondson grade, AFP and DFS data in AMC database, and the factors that might affect DFS in clinical data were preliminarily screened.

Liver subitem in the TCGA database consisted of 360 HCC cases with more than 9,000 mutant genes. Survival analysis of mutant genes was performed by survival package in R software, including 350 mutant genes in TCGA database and 172 mutant genes in AMC database. A total of 31 genes and 15 genes were screened from TCGA and AMC databases with log-rank P < 0.05, respectively. Then, the selected genes were cross-verified in the two databases.

### Analysis of race-related genes

The race information of 356 HCC cases in the TCGA database was completed. To study difference of genes among various races, we specifically distinguished Asian from non-Asian in the TCGA database. The Boruta algorithm in R software was used to preliminarily screen the genes with mutation differences between Asian and non-Asian HCC patients from the top 350 mutant genes of TCGA database. Chi-Square test (including Fisher’s test and Pearson’s test) was then used to screen the mutant genes preliminarily selected by feature selection for more accurate screening.

### Analysis of vascular invasion-related genes

The 306 HCC patients in the TCGA database had detailed vascular invasion information, including macrovascular invasion, microvascular invasion and non-vascular invasion. AMC database contained vascular invasion information of 231 HCC patients. The Inserm database contained vascular invasion information of 236 HCC patients. For the convenience of analysis, vascular invasion was divided into two categories, including vascular invasion and non-vascular invasion. First, Boruta algorithm, a feature selection algorithm in R software, was used to preliminarily screen the mutant genes that might have differences in vascular invasion from the 350 mutant genes of TCGA database, 172 mutant genes of AMC database and 211 mutant genes of Inserm database. Then, by Fisher’s test or Pearson’s test, P < 0.05 was taken to select the genes with different mutations between vascular invasion and non-vascular invasion.

### Whole-exome sequencing of 10 patients in our center and comparison with TCGA, AMC and Inserm databases

Tissue specimens were collected from 10 HCC patients who underwent liver resection in Nanjing Drum Tower Hospital from 2016 to 2017, paraffin-embedded sections were prepared, and whole-exome sequencing was performed (Shanghai Biotecan Pharmaceuticals Co., Ltd., Pudong New District, Shanghai, China). The study has agreement from the Institutional Ethics Review Board of Drum Tower Hospital and Nanjing University. All methods were performed in accordance with the relevant guidelines and regulations. The written informed consent was signed by all patients. The seven patients belonged to short-term recurrence group after radical surgery (recurrence time <6 months), and the other three cased showed recurrence after more than 6 months. The detailed pathological features of those 10 patients were shown in Fig. S3 and Table S5–7. The adapter and low-quality reads were removed from sequencing raw data, and the clean reads were aligned to the reference human genome (UCSC hg19) using the BurrowsWheeler Aligner. GATK and Picard tools were used for quality control, including duplicate removal, local realignment and generated quality statistics. Variants were annotated with ANNOVAR and the COSMIC database. For SNVs, only non-synonymous SNVs were taken into consideration, while the synonymous SNVs and SNVs in the non-coding region were removed. SNVs with more than 1% mutation frequency in 1,000 Genomes Project were excluded. In addition, GO enrichment and comparison with TCGA, AMC and Inserm databases were conducted to explore differences.

### The TCGA database was used to construct a recurrence prediction model based on mutant genes

The mutant gene data in TCGA database were used to build a model to predict the recurrence of patients, and then AMC data and the mutant gene data obtained from the whole-exome sequencing of 10 patients in our center were used for verification.

## Supplementary information

Supplementary Information.
