# Supplementary material for: Identification of a novel gene signature for the prediction of recurrence in HCC patients by machine learning of genome-wide databases
Source: Sci Rep. 2020 Mar 10;10:4435. doi: 10.1038/s41598-020-61298-3 (PMC7064516; doi:10.1038/s41598-020-61298-3)
Supplement: Supplementary file 1 — Supplementary Information. [file 41598_2020_61298_MOESM1_ESM.pdf]

Identification of a novel gene signature for the prediction of recurrence in HCC patients by machine learning of genome-wide databases

Jie Shen[1#], Liang Qi[1#], Zhengyun Zou[1#], Juan Du[1], Weiwei Kong[1], Lianjun Zhao[1], Jia Wei[1], Ling Lin[2], Min Ren[2], Baorui Liu[1]*

[1] *Comprehensive Cancer Centre of Drum Tower Hospital, Medical School of Nanjing University, Clinical Cancer Institute of Nanjing University, Nanjing 210008, Jiangsu Province, China*

[2] *Shanghai Biotecan Pharmaceuticals Co., Ltd., Pudong New District, Shanghai, China*

[#] These authors contributed equally to this work.

***Correspondence to**: Bao-rui Liu, MD, Ph. D, Comprehensive Cancer Centre of Drum Tower Hospital, Medical School of Nanjing University, Clinical Cancer Institute of Nanjing University, 321 Zhongshan Road, Nanjing 210008, Jiangsu Province, China.

Email: baoruiliu@nju.edu.cn

Tel.: +86-25-83106666-61331

Fax: +86-25-83105082

Figure S1.A: DNAH5 mutation and DFS in TCGA database; B: DNAH5 mutation and DFS in AMC; C: ERBB4 mutation and DFS in TCGA database; D: ERBB4 mutation and DFS in AMC; E: ABCA12 mutation and DFS in TCGA database; F: ABCA12 mutation and DFS in AMC; G: ROBO2 mutation and DFS in TCGA database; H: ROBO2 mutation and DFS in AMC.

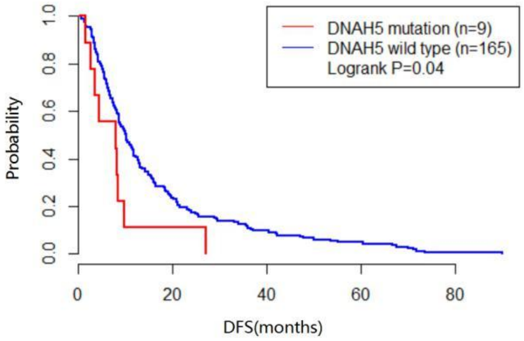

A

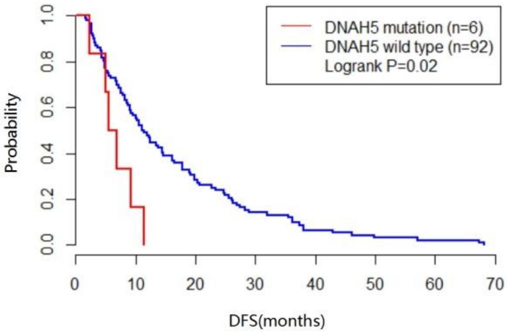

B

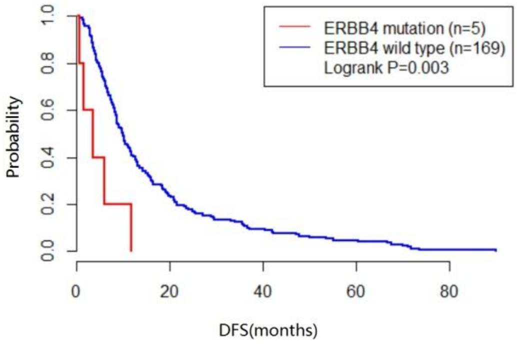

C

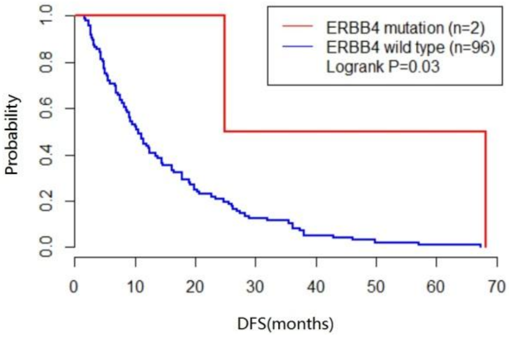

D

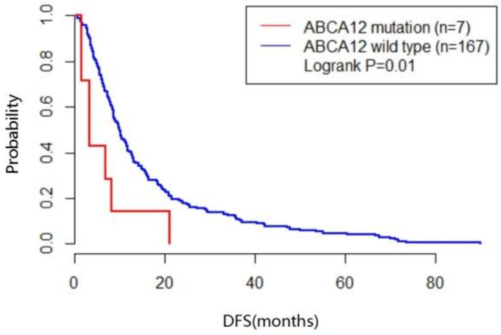

E

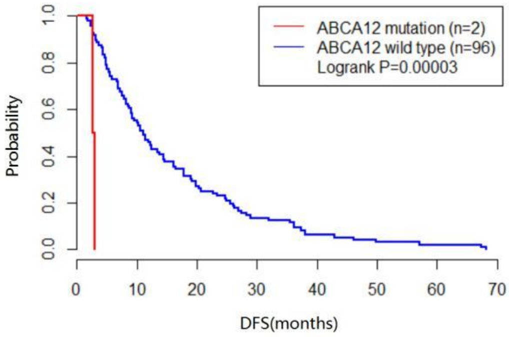

F

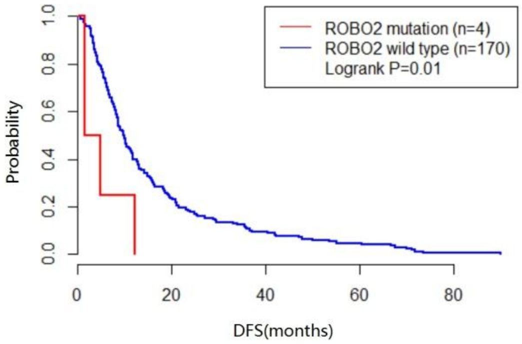

ROBO2 mutation (n=4)
ROBO2 wild type (n=170)
Logrank P=0.01
Probability
1.0
0.8
0.6
0.4
0.2
0.0
0
20
40
60
80
DFS(months)

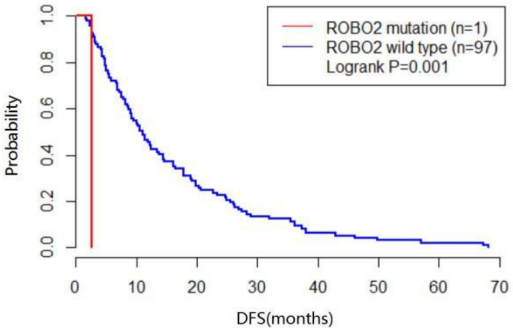

ROBO2 mutation (n=1)
ROBO2 wild type (n=97)
Logrank P=0.001
Probability
1.0
0.8
0.6
0.4
0.2
0.0
0
10
20
30
40
50
60
70
DFS(months)

G

H

Figure S2. The data of HCC patients listed in the TCGA database and those included in our study. The original data downloaded from http://www.cbioportal.org.

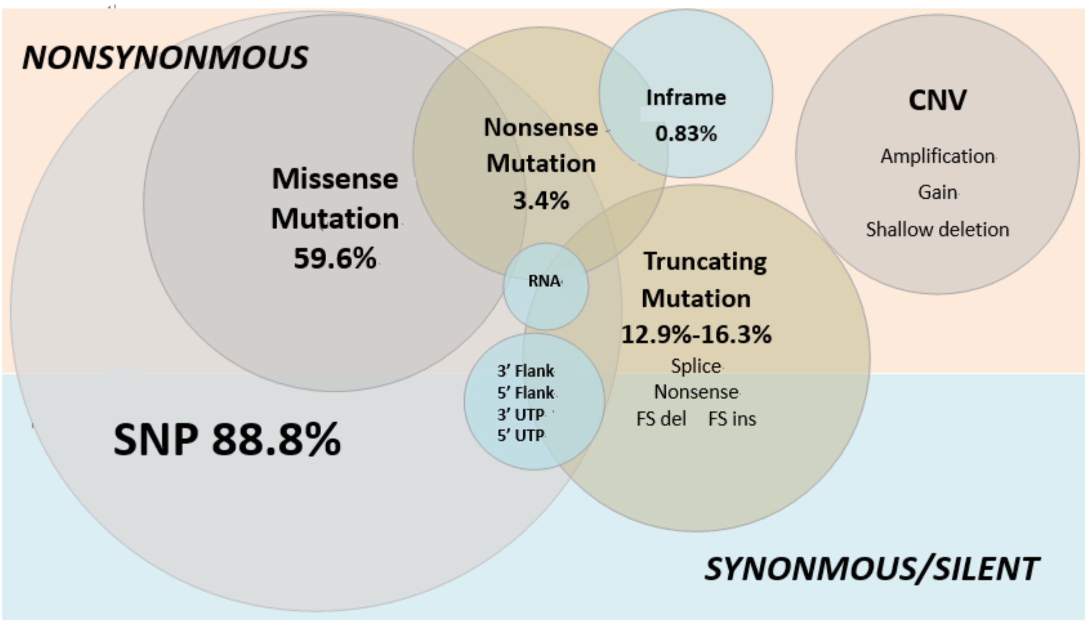

Figure S3. Pathological HE staining of 10 HCC patients. A: Patient1 (HE, 2,025X), B: Patient2 (HE, 1,214X), C: Patient3 (HE, 1,854X), D: Patient4 (HE, 1,744X), E: Patient5 (HE, 1,755X), F: Patient6 (HE, 1,143X), G: Patient7 (HE, 1,318X), H: Patient8 (HE, 2,347X), I: Patient9 (HE, 1,934X), J: Patient10 (HE, 2,157X).

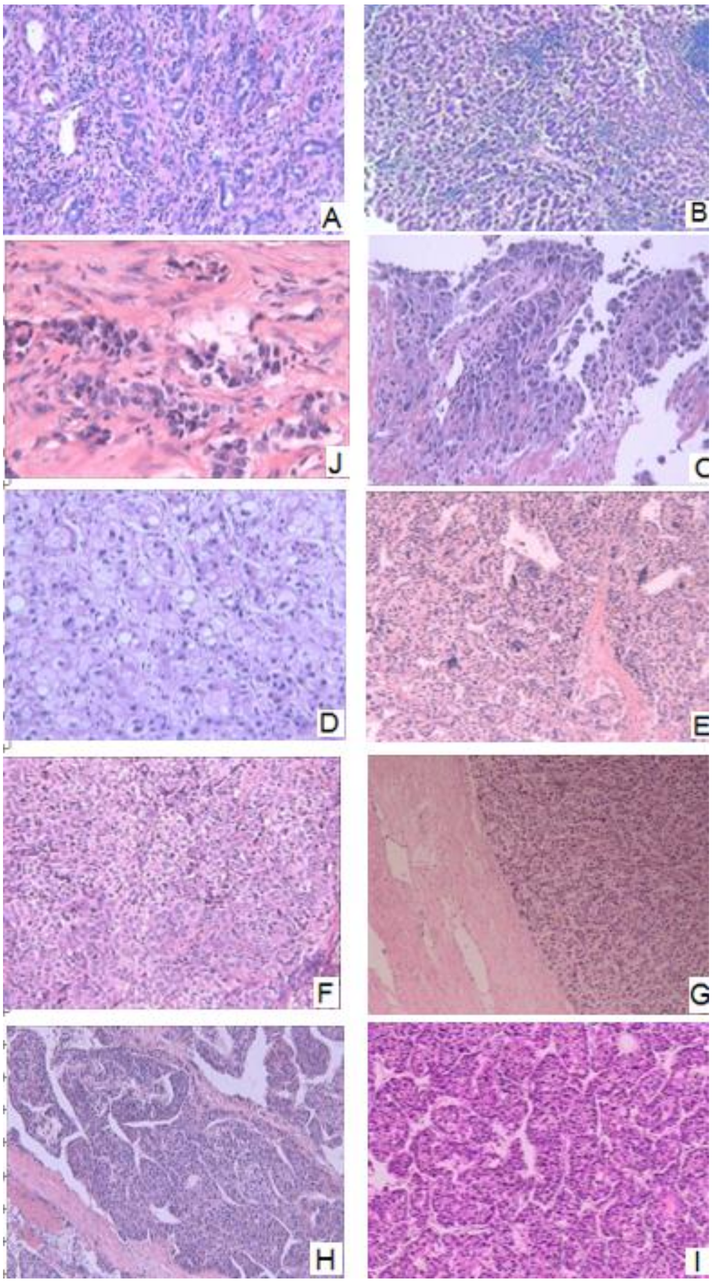

TableS1. Thelistofthe 127 genesfrom TCGA database

127 Genes							
TTN	ALMS1	KMT2D	PKHD1L1	DMD	SVEP1	MT-ND6	ROBO2
TP53	HMCN1	SYNE1	FREM2	MT-CO1	HERC1	NEFH	
CTNNB1	FRAS1	DSPP	MYO3A	KEAP1	LRP2	NFE2L2	
MUC16	USH2A	BAP1	DNAH5	GCN1	FBN1	SPEG	
ALB	MUC4	FAT4	DYNC2H1	HTT	PKHD1	FANCM	
PCLO	FLG	CUBN	DOCK2	KIAA1109	UNC79	KMT2A	
APOB	AHNAK2	DNAH9	RYR3	PTPRQ	DCHS1	MAP1B	
RYR2	NBEA	SYNE2	MUC2	FBN2	POLQ	SACS	
ND5	EYS	TCHH	HERC2	PREX2	DNAH2	DNAH17	
CSMD3	CSMD1	ZNF469	DNAH10	FMN2	LAMA1	COL6A6	
OBSCN	AXIN1	HSPG2	MUC17	CSMD2	PREX1	HECTD4	
ABCA13	RB1	ZFHX4	LRP1	FASN	NEB	JAK1	
ARID1A	DNAH7	UNC80	ANKRD12	FAT2	MYCBP2	NCAM1	
CACNA1E	ADGRV1	COL11A1	ABCA12	COL6A3	UNC13C	MYO18B	
LRP1B	CYTB	BIRC6	DCHS2	COL12A1	DSCAM	ITPR1	
XIRP2	ARID2	KMT2B	PRUNE2	ATR	SDK1	ASCC3	
SPTA1	DNAH6	WDR87	KMT2C	SETD2	PCDH15	DST	
RYR1	FAT3	AHNAK	DNAH8	MDN1	KIF26B	TENM4	

Table S2. Classification of result confusion matrix of decision tree

The true situation	Prediction result		class precision (specificity)	AMC data validation Accuracy
	Relapse within 6 months	More than 6 mouths relapse		
Relapse within 6 months	**2**	**3**	40.00%	74.19% (TCGA)
More than 6 mouths relapse	**5**	**21**	80.77%	
Class recall (sensitivity)	28.57%	87.50%		
Relapse within 6 months	**1**	**27**	3.57%	70.41% (AMC)
More than 6 mouths relapse	**2**	**68**	97.14%	
Class recall (sensitivity)	33.33%	71.58%		

Table S3. Classification of result confusion matrix of SVM (accuracy: 80.65%)

The true situation	Prediction result		class precision (specificity)
	Relapse within 6 months	More than 6 mouths relapse	
Relapse within 6 months	**1**	**0**	100.00%
More than 6 mouths relapse	**6**	**24**	80.00%
class recall(sensitivity)	14.29%	100.00%	

TableS4. Conformance of the test model in 10 HCC patients

Patient	DFS	**NEB**	**ATR**	**FREM2**	**TTN**	**ALB**	**XIRP2**	**RYR1**	conformation
Patient1	2.5m	**0**	**0**	**0**	**0**	**0**	**1**	**0**	✖
Patient2	2.5m	**1**	**0**	**0**	**1**	**0**	**0**	**1**	✔
Patient3	3.0m	**0**	**0**	**0**	**1**	**0**	**0**	**0**	✖
Patient4	>6.0m	**0**	**0**	**0**	**1**	**0**	**1**	**0**	✔
Patient5	>6.0m	**0**	**0**	**0**	**1**	**1**	**0**	**0**	✔
Patient6	3.0m	**1**	**0**	**1**	**0**	**0**	**0**	**1**	✔
Patient7	>6.0m	**0**	**0**	**0**	**0**	**0**	**0**	**0**	✔
Patient8	3.0m	**0**	**1**	**0**	**1**	**0**	**1**	**0**	✔
Patient9	5.0m	**1**	**0**	**0**	**1**	**0**	**0**	**0**	✔
Patient10	5.0m	**1**	**0**	**0**	**0**	**0**	**0**	**0**	✔

(“1”indicates that the gene is mutated)

Table S5. Histopathologic staging and liver cirrhosis grade of 10 HCC patients

Patient	Sex	Age	DFS	Peripheral hepatic steatosis grade	Inflammatory grade of peripheral liver tissue	Fibrosis grading	AJCC（7th）	T	N	M
Patient1	Male	62	2.5m	F0	G3	S4	IV	T3b	N1	M0
Patient2	Male	46	2.5m	F0	G2	S3-4	II	T2	N0	M0
Patient3	Male	46	3.0m	F0	G3	S3-4	II	T2	N0	M0
Patient4	Male	54	> 6.0m	F1	G2	S2-3	IIIB	T3b	N0	M0
Patient5	Male	49	> 6.0m	F0	G3	S3-4	IIIB	T3b	N0	M0
Patient6	Male	40	3.0m	F0	G3	S3-4	IIIC	T4	N0	M0
Patient7	Male	47	> 6.0m	F0	G1	S2-3	IIIA	T3a	N0	M0
Patient8	Male	60	3.0m	F0	G2	S3	II	T2	N0	M0
Patient9	Male	60	5.0m	F0	G3	S3	I	T1	N0	M0
Patient10	Female	39	5.0m	F0	G2	S2	II	T2	N0	M0

Table S6. Immunohistochemistry of 10 HCC patients

Patient	GPC-3	CK19	Ki-67	GLS1	AFP	VEGFR2	PD1	Hep1	CK7	CD10	CD56	Sall4	Arg-1	GS	βcatenin	PD-L1
Patient1	—	+	40%+	3+	—	2+	—	2+	+	1+	—	—	—	—	—	—
Patient2	3+	—	20%+	+	3+	3+	3+	—	—	—	—	—	—	—	+	3+
Patient3	1+	—	30%+	1+	+	1+	—	—	—	—	—	—	—	—	—	—
Patient4	+	—	30%+	+	—	—	—	—	+	—	—	—	1+	1+	—	—
Patient5	+	—	30%+	+	—	1+	—	—	—	—	—	—	—	—	—	—
Patient6	—	1+	40%+	1+	—	—	—	—	2+	—	—	—	—	—	—	—
Patient7	—	—	20%+	1+	—	—	—	—	—	—	—	—	—	—	—	—
Patient8	2+	—	40%+	2+	3+	—	—	—	—	1+	—	—	—	3+	—	1+
Patient9	1+	—	15%+	2+	—	—	—	—	—	—	—	—	—	—	—	—
Patient10	1+	—	15%+	2+	—	1+	—	—	—	—	—	—	—	—	—	—

Table S7. Gross and pathological features of the tumors in 10 HCC patients

Patient	Tumor number	Tumor Size	Histological classification	Edmondson-Steiner grade	Portal vein and its main branches carcinoma thrombus	Intracholangial carcinoma thrombus	Nerve invasion	Micro vascular invasion (MVI)
Patient1	1	4.0×3.7×3.5	Solid and pseudoadenoid	III	Yes	No	No	1
Patient2	1	2.0×2.0×1.3	Beam type	II-III	No	No	No	1
Patient3	1	4.5×4.0×3.0	Beam type	II-III	No	No	No	1
Patient4	3	2.5×2.5×2.0	Beam and pseudoadenoid	II-III	Yes	Yes	No	1
Patient5	1	7.5×6.0×5.0	Beam type	III	Yes	No	No	2
Patient6	1	5.5×4.5×4.0	Beam type	III	No	No	No	1
Patient7	3	16.0×14.0×11.5	Beam type	II	No	No	No	0
Patient8	1	2.3×1.8×1.3	Beam and pseudoadenoid	II-III	No	No	No	1
Patient9	1	15.0×13.0×11.0	Beam type	II	No	No	No	0
Patient10	3	3.0×3.0×4.0	Beam type	II-III	No	No	No	0
